# Prospektive Verlaufsbeobachtung einer universitären Rheumaambulanzkohorte während der ersten Welle der COVID-19-Pandemie: Welche Lehren kann man ziehen?

**DOI:** 10.1007/s00393-020-00935-8

**Published:** 2020-11-30

**Authors:** M. C. Braunisch, Q. Bachmann, A. Hammitzsch, G. Lorenz, F. Geisler, C. Schmaderer, U. Heemann, P. Moog

**Affiliations:** 1 Sektion Rheumatologie, Abteilung für Nephrologie, II. Medizinische Klinik, Klinikum rechts der Isar, Fakultät für Medizin, Technische Universität München, Ismaninger Str. 22, 81675 München, Deutschland; 2 II. Medizinische Klinik, Klinikum rechts der Isar, Fakultät für Medizin, Technische Universität München, München, Deutschland

**Keywords:** SARS-CoV-2, COVID-19-Pandemie, Rheumaerkrankung, Immunsuppressive Therapie, Therapieadjustierung, SARS-CoV-2, COVID-19 pandemic, Rheumatic disease, Immunosuppressive medication, Treatment adjustment

## Abstract

**Hintergrund:**

Im März 2020 breitete sich die SARS-CoV-2-Pandemie initial v. a. in Bayern aus. Zu diesem Zeitpunkt war weitgehend unklar, wie mit der immunmodulatorischen Therapie bei Rheumapatienten umzugehen ist.

**Ziel der Arbeit:**

Das Ziel war es, den Einfluss der Pandemie auf klinische Entscheidungen zu erfassen.

**Material und Methoden:**

Es wurden zwischen dem 16.03. und 31.07.2020 Patienten eingeschlossen, die sich in der Rheumaambulanz des Klinikums rechts der Isar vorstellten. Anpassungen der Therapie erfolgten nach klinischem Ermessen und in Anlehnung an die Handlungsempfehlungen der DGRh.

**Ergebnisse:**

Es wurden 322 Patienten eingeschlossen. Die häufigsten Diagnosen waren die rheumatoide Arthritis mit 17 %, die ANCA-assoziierte Vaskulitis (AAV) mit 14 % sowie der SLE mit 12 %; 262 Patienten erhielten eine DMARD-Therapie und 77 Patienten orale Glukokortikoide. Es lagen 5 SARS-CoV-2-Verdachtsfälle vor. Kein Patient erkrankte nachweislich an COVID-19. Eine Therapieänderung erfolgte aufgrund der Pandemie bei 40 Patienten. Dabei kam es bei 3 Patienten zu einem Flare der Grunderkrankung. Eine Therapiedeeskalation erfolgte am häufigsten bei AAV, IgG4-assoziierter Erkrankung sowie bei gleichzeitig bestehenden Malignomen und beim Einsatz von Rituximab.

**Diskussion:**

In dieser Single-Center-Kohorte legt das gänzliche Fehlen von nachweislichen SARS-CoV-2-Infektionen in einer sonst relativ stark betroffenen Region den Schluss nahe, dass kein überproportional erhöhtes Infektionsrisiko für Patienten mit entzündlich rheumatischen Erkrankungen zu bestehen scheint. Eine Fortführung der meisten immunsuppressiven Therapien erscheint daher sinnvoll.

## Abstract (en)

**Background:**

In March 2020 the SARS-CoV‑2 pandemic disseminated initially especially in Bavaria. At that time data on patients with rheumatic diseases and immunomodulatory treatment was lacking.

**Objective:**

The aim was to analyze the influence of the SARS-CoV‑2 pandemic on the clinical treatment strategy.

**Material and methods:**

Between 16 March and 31 July 2020 all patients who consecutively presented at the rheumatology outpatient clinic of the Klinikum rechts der Isar of the Technical University of Munich were included in the study. Individual treatment adjustments were based on clinical judgment and the recommendations for action of the German Society for Rheumatology (DGRh).

**Results:**

A total of 322 patients were included. The most frequent diagnosis was rheumatoid arthritis with 17%, ANCA-associated vasculitis (AAV) with 14% and SLE with 12%. Of the patients 262 were on DMARD treatment and 77 received oral glucocorticoids. There were 5 cases of suspected SARS-CoV‑2 infection; however, no patient verifiably became ill due to COVID-19. In 40 patients, treatment adjustments were done due to the pandemic, whereby 3 patients developed a flare of the underlying disease. In retrospect, treatment de-escalation occurred most frequently in AAV, IgG4-related disease, immunosuppressive treatment with rituximab and the simultaneous presence of malignant diseases.

**Conclusion:**

The total lack of confirmed SARS-CoV‑2 infections in an otherwise strongly affected region could indicate that the infection risk for SARS-CoV‑2 is not substantially increased for patients with inflammatory rheumatic diseases. A continuation of most immunosuppressive medications therefore seems reasonable during the ongoing pandemic.

Im Dezember 2019 ist in Wuhan in der Provinz Hubei in China ein neuartiges Coronavirus mit dem Namen „severe acute respiratory syndrome coronavirus 2“ (SARS-CoV-2) identifiziert worden, welches die Corona-Virus-Krankheit-2019 (COVID-19) verursacht [1, 2]. Wie auch bei vorangegangenen humanpathogenen Corona-Virus-Epidemien (SARS-CoV-1 2002–2003, Middle East Respiratory Syndrome MERS seit 2012) wurde frühzeitig klar, dass ein Teil der Patienten sehr fulminante Pneumonien mit Hyperinflammation und akutem Lungenversagen (acute respiratory distress syndrome/ARDS) entwickelt [1–3]. Am 11.03.2020 stufte die Weltgesundheitsorganisation die initial auf China begrenzte Epidemie zur Pandemie hoch. Im März 2020 kam es zu einem starken Anstieg der Infektionen in Deutschland, welcher initial v. a. aufgrund verschiedener Umstände von einem Anstieg der Fallzahlen in Bayern getrieben wurde. Zu diesem Zeitpunkt stellte sich das gesamte Gesundheitssystem auf eine Flut von teilweise schwersterkrankten Patienten ein. Aufgrund dessen erfolgte am 16.03.2020 die Ausrufung des Katastrophenfalls in Bayern. In einem Hochinzidenzgebiet mussten wir uns insbesondere fragen, ob die von uns betreuten Patienten durch ihre Grunderkrankung selbst oder deren meist immunsuppressive oder immunmodulatorische Therapie zu einer besonderen Risikogruppe zu zählen sind. Es lagen jedoch nur sehr wenige Daten zu immunsupprimierten Patienten vor. Die bis dahin größte veröffentlichte Studie (*n* = 1099) aus China beinhaltete nur *n* = 2 (0,2 %) Patienten mit einer Immundefizienz [1]. Neben den logistischen Problemen der regelmäßigen Kontrollen und Rezeptierung der antirheumatischen Medikamente während des bundesweiten Lockdowns war eine entscheidende Frage zu Beginn der Pandemie folgende: Was ist schlechter für den Patienten? Eine COVID-19-Erkrankung unter einer fortgesetzten immunsuppressiven DMARD(„disease modifiying antirheumatic drug“)-Therapie oder eine COVID-19-Erkrankung in einer Situation, in der wir durch eine Therapiepause möglicherweise einen Schub der Grunderkrankung provozieren, was uns zu einer mittelhoch oder hoch dosierten Glukokortikoidtherapie zwingen würde? In beeindruckender Geschwindigkeit haben zur Klärung dieser Frage nationale und internationale wissenschaftliche Allianzen Register für rheumatologische Patienten mit SARS-CoV-2-Infektion ins Leben gerufen, und die ersten Publikationen daraus liegen bereits vor [4–7]. Während die verschiedenen Register den Fokus klar auf die infizierten Patienten richten, lag unser Interesse neben der Inzidenz und dem Verlauf der Infektion in unserer Kohorte auch darauf, wie mit nicht an COVID-19 erkrankten Patienten hinsichtlich der Fortführung ihrer Therapie verfahren wird.

## Material und Methoden

### Patientenkohorte

Ab dem Erhalt des Ethikvotums am 16.03.2020 wurden alle Patienten, die sich konsekutiv in der Rheumaambulanz des Universitätsklinikums rechts der Isar vorstellten, nach Aufklärung und Einwilligung in die Studie eingeschlossen. Es erfolgte eine prospektive nicht-interventionelle Verlaufsbeobachtung. Ausschlusskriterien waren eine fehlende Einverständniserklärung sowie Alter <18 Jahre.

Klinische Informationen wie die Medikation, Komorbiditäten und Impfstatus wurden anhand der schriftlichen Dokumentation erhoben und bei Einschluss erfragt. Zudem wurde erfasst, falls eine SARS-CoV-2-Polymerasekettenreaktion (PCR) oder Serologie durchgeführt wurde. Als Lungenbeteiligung wurde der historische Befall der Lunge durch die rheumatologische Grunderkrankung definiert.

### Verlaufsbeobachtung

Patienten wurden weiterhin in der Regel mindestens alle 12 Wochen zu den regulären Ambulanzterminen kontaktiert, und neben dem Assessment der rheumatologischen Krankheitsaktivität auch bezüglich COVID-19-Symptomen befragt (Krankheitsgefühl, Fieber, Schüttelfrost, konjunktivale Injektion, Rhinitis, Kopfschmerzen, Husten, Halsschmerzen, Auswurf, Hämoptoe, Dyspnoe, Schwindel, Myalgien, Gliederschmerzen, abdominelle Schmerzen, Übelkeit, Erbrechen, Durchfall, Appetitlosigkeit, Geschmacksverlust, Gewichtsverlust) [1, 2]. Zudem wurden Patienten dazu angehalten, sich unverzüglich bei Verdacht auf eine COVID-19-Erkrankung zu melden. Hierzu wurden erweiterte telefonische Sprechzeiten mit Arztkontakt eingerichtet. Risikofaktoren für einen schweren Verlauf wurden gemäß Zhen et al. 2020 definiert [8].

### Therapiestrategie

Individuelle Anpassung der Therapie erfolgte initial anhand der von der deutschen Gesellschaft für Rheumatologie (DGRh) am 25.03.2020 online und im Mai 2020 in der Zeitschrift für Rheumatologie veröffentlichten Handlungsempfehlungen [9]. Eine Therapiereduktion oder -beendigung erfolgte nur bei Patienten mit einem stabilen Krankheitsverlauf. Zudem wurde die fachspezifische Literatur aufmerksam nach Daten bei Rheumapatienten verfolgt und unsere Therapiestrategie entsprechend angepasst [4–6].

### Outcome-Parameter und statistische Analyse

Als primäre Zielgröße wurden das Auftreten einer COVID-19-Erkrankung sowie der kombinierte Endpunkt Behandlung auf Intensivstation oder Tod definiert. Sekundäre Zielgrößen waren die Schwere des Verlaufs einer COVID-19-Erkrankung sowie die Veränderung der immunmodulatorischen Therapie unter Pandemiebedingungen. Kategoriale Daten werden als absolute und relative Häufigkeiten angegeben. Kontinuierliche Variablen werden, da nicht normalverteilt, als Median und Interquartilsabstand berichtet. Gruppenvergleiche wurden mittels Chi-Quadrattest berechnet. Zur Analyse von Faktoren, die retrospektiv mit einer Therapieveränderung bei Patienten unter Therapie assoziiert sind, wurden 3 logistische Regressionen mit Rückwärtseinschluss durchgeführt. Modell A enthielt die häufigsten Diagnosen der Kohorte, Modell B die DMARD-Medikationen sowie eine Prednisolon-Dosis ≥10 mg/Tag und Modell C Alter, Nikotinabusus und die Komorbiditäten. Alle statistischen Tests waren zweiseitig und *p*-Werte <0,05 wurden als signifikant erachtet. Die statistische Analyse erfolgte mittels SPSS Version 26.0 (SPSS, Inc., Chicago, IL, USA).

## Ergebnisse

### Patientencharakteristika

Während der ersten Pandemiewelle wurden konsekutiv 322 Patienten zwischen dem 16.03. und 31.07.2020 eingeschlossen. Zehn Patienten stimmten einer Teilnahme nicht zu. Das mediane Alter der Kohorte lag bei 57,3 Jahren; 107 (33,3 %) der Patienten waren männlich. Der mediane Body-Mass-Index betrug 25,4 kg/m^2^; 258 der 322 (80,1 %) Patienten waren in Remission ihrer rheumatologischen Grunderkrankung. Eine Lungenbeteiligung aufgrund der rheumatischen Grunderkrankung lag bei 68 (21,1 %) der Patienten vor; 51 (15,8 %) Patienten waren aktive Raucher. Eine aktuelle Influenzaimpfung 2019/2020 lag bei 154 (47,8 %), eine Pneumokokkenimpfung bei 130 (40,4 %) der Patienten vor. Zwei Patientinnen waren zur Baselineerhebung schwanger. Eine Osteoporose bestand bei 26 (8,1 %), eine Osteopenie bei 21 (6,5 %) der Patienten. Für weitere Details s. Tab. 1.

**Table 1:** 

*Geschlecht, männlich*	107 (33,3 %)
*Alter, Jahre*	57,3 (42,0–66,5)
*Body-Mass-Index, kg/m*^*2*^	25,4 (22,1–29,2)
*In Remission*	258 (80,1 %)
*Lungenbeteiligung durch Grunderkrankung*	68 (21,1 %)
*Schwangerschaft*	2 (0,6 %)
*Diagnosen der häufigsten Rheumaerkrankungen*	
Rheumatoide Arthritis	57 (16,7 %)
Granulomatose mit Polyangiitis und mikroskopische Polyangiitis	46 (13,5 %)
Systemischer Lupus erythematodes mit oder ohne APS	42 (12,3 %)
Spondylarthritis	29 (8,5 %)
Psoriasisarthritis	24 (7,0 %)
Großgefäßvaskulitis	17 (5,0 %)
Eosinophile Granulomatose mit Polyangiitis	15 (4,4 %)
IgG4-assoziierte Erkrankung	14 (4,1 %)
Systemsklerose	14 (4,1 %)
Sjögren-Syndrom	13 (3,8 %)
Sarkoidose	13 (3,8 %)
Autoinflammationssyndrome	8 (2,3 %)
Inflammatorische Myopathien	5 (1,5 %)
Panarteriitis nodosa	2 (0,6 %)
Andere Erkrankungen	43 (12,6 %)
*Doppeldiagnosen*	20
*DMARD-Medikation*	
Keine DMARD-Medikation	60 (15,5 %)
csDMARD	131 (33,8 %)
TNF-Blocker	50 (12,9 %)
Rituximab	48 (12,4 %)
Hydroxychloroquin	39 (10,1 %)
tsDMARD	17 (4,4 %)
IL-6-Rezeptor Blocker	14 (3,6 %)
IL-17-Blocker	12 (3,1 %)
IL-5-Blocker	6 (1,5 %)
IL-1-Blocker	4 (1,0 %)
Andere Medikation	7 (1,8 %)
*Prednisolon*	77 (23,9 %)
*Influenzaimpfung 2019/2020*	154 (47,8 %)
*Pneumokokkenimpfung*	130 (40,4 %)
*Nikotinabusus*	
Aktueller Nikotinabusus	51 (15,8 %)
Nikotinabusus in den letzten 10 Jahren	24 (7,4 %)
*Asthma bronchiale*	30 (9,3 %)
*Chronisch obstruktive Lungenerkrankung*	6 (1,9 %)
*Pulmonalarterielle Hypertonie*	7 (2,2 %)
*Diabetes mellitus*	36 (11,2 %)
*Arterielle Hypertonie*	117 (36,3 %)
*Kardiovaskuläre Erkrankung*	66 (20,5 %)
*Zerebrovaskuläre Erkrankung*	27 (8,4 %)
*Infektiologische Erkrankung*	14 (4,3 %)
*Maligne Erkrankung*	27 (8,4 %)
*Chronische Nierenerkrankung*	43 (13,3 %)
*Chronische Lebererkrankung*	3 (0,9 %)

Infektiologische Erkrankung beinhaltet HIV, Virushepatitis, parasitäre Erkrankungen

*APS* Antiphospholipidsyndrom, *IL* Interleukin, *IgG* Immunglobulin G, *DMARD* „disease modifiying antirheumatic drug“, *tsDMARD* „targeted systemic DMARD“, *csDMARD* konventionell synthetisches DMARD (Azathioprin, Cyclophosphamid, Cyclosporin, Leflunomid, Methotrexat, Mycophenolat-Mofetil/Mycophenolsäure, Sulfasalazin, Tacrolimus), *TNF* Tumor-Nekrose-Faktor

Die Diagnosen sowie die Medikation der Kohorte sind in Tab. 1 dargestellt. Die häufigsten Diagnosen waren die rheumatoide Arthritis mit 16,7 %, die ANCA-assoziierten Vaskulitiden Granulomatose mit Polyangiitis (GPA) und mikroskopische Polyangiitis (MPA) mit 13,5 %, sowie der systemische Lupus erythematodes (mit oder ohne Antiphospholipidsyndrom) mit 12,3 %. Von den 322 Patienten erhielten 60 (18,6 %) keine Therapie, 177 (55,0 %) 1, 68 (21,1 %) 2 und 5 (1,6 %) 3 DMARDs; 77 (23,9 %) Patienten erhielten orale Glukokortikoide mit einer medianen Prednisolon-Dosis von 6,8 mg/Tag (2,5–8,8).

Die Komorbiditäten sind in Tab. 1 aufgelistet. Die häufigsten Komorbiditäten waren eine arterielle Hypertonie bei 117 (36,3 %), eine kardiovaskuläre Erkrankung bei 66 (20,5 %), eine chronische Niereninsuffizienz bei 43 (13,3 %) sowie ein Diabetes mellitus bei 36 (11,2 %) der Patienten.

### Strukturelle Anpassungen der ambulanten Versorgung

Die ambulante rheumatologische Versorgung wurde vom 16.03. bis zum 20.05.2020 in allen möglichen Fällen auf eine telefonische Sprechstunde umgestellt. Nur in Notfällen wurde ein Präsenztermin vereinbart. Von den 39 Patienten mit Hydroxychloroquin-Therapie kam es bei 4 zu einem passageren Lieferengpass Anfang April 2020. Nach telefonischer Kontaktaufnahme mit der Apotheke nach den Vorgaben der DGRh (Vermerk der Diagnose auf dem Rezept, Verweis auf Sonderkontingent nach Absprachen der DGRh mit bestimmten Pharmaunternehmen) konnte dieser problemlos überbrückt werden. Zudem war bei einem Patienten Mitte April Trimethoprim/Sulfamethoxazol passager nicht lieferbar.

### COVID-19-Erkrankung

Die primäre Zielgröße und der primäre kombinierte Endpunkt ebenso wie der sekundäre Endpunkt zur Analyse der Schwere des Verlaufs wurden nicht erreicht. Innerhalb der Verlaufsbeobachtung bestand bei 5 Patienten der Verdacht auf eine SARS-CoV-2-Infektion. Die Symptome dieser Patienten sind in Tab. 2 aufgelistet. Eine stationäre Aufnahme wurde bei keinem dieser Patienten notwendig. Die Tab. 3 listet die Diagnosen, die Medikation sowie die Therapieänderungen dieser 5 Verdachtsfälle auf. Testungen auf SARS-CoV‑2, die bei 3 Patienten durchgeführt wurden, zeigten ein negatives Ergebnis (Tab. 2). Zwei Patienten wurden nach Aussagen der Patienten nicht getestet. Beim ersten bestanden berufliche Kontakte zu in China lebenden Personen. Der Patient erkrankte Anfang Februar an einem fieberhaften Infekt. Der Hausarzt stellte die Diagnose eines Virusinfekts, jedoch wurde wahrscheinlich keine COVID-19-Erkrankung vermutet und deshalb keine Testung veranlasst. Der zweite Patient berichtete Mitte März in unserer Telefonsprechstunde von typischen Symptomen. Eine Testung wurde dringend angeraten, jedoch nicht wahrgenommen.

**Table 2:** 

Symptom	Häufigkeit
Krankheitsgefühl	5
Fieber (>38 °C)	4
Kopfschmerzen	3
Husten	3
Halsschmerzen	3
Gliederschmerzen	2
Diarrhö	3
Geschmacksverlust	1
*Therapie*	
Ambulant, symptomatisch	5
*Bisherige Testungen (negativ)*	3
PCR	1
Serologie	2
*Datum des Verdachtsfalls*	04.02., 15.03., 16.03., 17.03., 24.03.2020

**Table 3:** 

Patient	Diagnose	Medikation	Therapieänderung
1	Reaktive Arthritis	15 mg/Tag Prednisolon, SSZ	Prednisolon-Reduktion auf 5 mg/Tag
2	Rheumatoide Arthritis	MTX, Golimumab	–
3	IgG4-assoziierte Erkrankung	–	–
4	Axiale Spondylarthritis	Secukinumab	Dosisintervallverlängerung
5	Sjögren-Syndrom	MTX, HCQ	MTX pausiert

*MTX* Methotrexat, *HCQ* Hydroxychloroquin, *SSZ* Sulfasalazin

**Figure 1:**
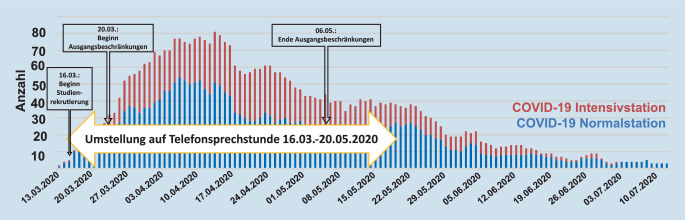

Es erfolgten 6 PCR-Nasen‑/Rachenabstriche sowie 33 serologische SARS-CoV-2-Testungen. Davon bestand nur bei 1 Patientin mit IgG4-assoziierter Erkrankung ein positiver unspezifischer Nachweis von IgM bei vollkommener Symptomfreiheit. Die Wiederholung der serologischen Testung erbrachte ca. 3 Monate später einen unauffälligen Befund bei dieser Patientin. Risikofaktoren für einen schweren oder tödlichen Verlauf sind in Tab. 4 aufgelistet. Im gleichen Zeitraum wurden am Klinikum rechts der Isar 223 Patienten wegen COVID-19 stationär behandelt, davon 84 auf Intensivstation, 27 Patienten verstarben (Abb. 1).

**Table 4:** 

Risikofaktor	Gesamtkohorte (*n* = 322)	Patienten unter Therapie		
		Ohne Therapieveränderung (*n* = 262)	Mit Therapieveränderung (*n* = 40)	*P*
Alter ≥65 Jahre	92 (28,6 %)	61 (27,4 %)	15 (37,5 %)	0,19
Männliches Geschlecht	107 (33,2 %)	71 (31,8 %)	15 (37,5 %)	0,47
Aktueller Nikotinabusus	51 (15,8 %)	52 (23,3 %)	10 (25,0 %)	0,84
Arterielle Hypertonie	117 (36,3 %)	73(32,7 %)	19 (47,5 %)	0,075
Kardiovaskuläre Erkrankung	66 (20,5 %)	49 (22,0 %)	6 (15,0 %)	0,40
Pulmonale Erkrankung	36 (11,2 %)	27 (12 %)	5 (12,5 %)	1,00

Pulmonale Erkrankung beinhaltet Asthma bronchiale und chronisch obstruktive Lungenerkrankung

### Therapieveränderungen

**Figure 2:**
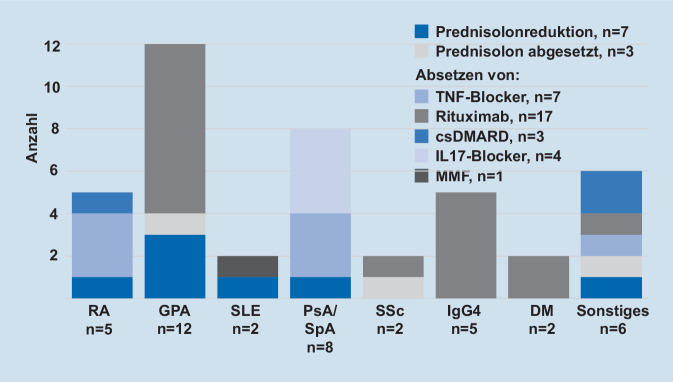

Bei 222 (84,7 % aller Patienten mit vorbestehender Therapie) Patienten wurde die Therapie ohne Änderungen fortgeführt. Aufgrund der Pandemie veränderten (Reduktion oder Absetzen) wir die immunmodulatorische Therapie bei 40 (15,3 %) Patienten (Abb. 2). Bei 10 (25,0 %) dieser 40 Patienten erfolgte eine mediane Prednisolon-Reduktion von 5,0 (1,5–10,0) auf 2,5 (0,0–5,0) mg. Von den 50 Patienten mit einem medianen Alter von 43,9 (37,8–59,6) Jahren, die einen TNF-Inhibitor erhielten, wurde dieser bei 7 Patienten bei klinischer Remission der Grunderkrankung und aufgrund der Pandemie abgesetzt. Von den 48 Patienten mit einem medianen Alter von 64,9 (52,7–74,1) Jahren, die Rituximab erhielten, wurde dieses bei 17 Patienten abgesetzt.

Bei 3 Patientinnen in Remission kam es nach Therapiereduktion/-pause folgender Medikamente zu einem Flare der jeweiligen Grunderkrankung: Secukinumab bei einer Patientin mit Psoriasisarthritis; Secukinumab bei einer Patientin mit axialer Spondylarthritis und Rituximab bei einer Patientin mit IgG4-assoziierter Erkrankung. Es fanden sich keine signifikanten Gruppenunterschiede für Risikofaktoren für einen schweren oder tödlichen Verlauf zwischen den Patienten mit und ohne Therapieveränderungen (Tab. 4). In den 3 logistischen Regressionen zeigten sich von den rheumatischen Grunderkrankungen die GPA/MPA sowie die IgG4-assoziierten Krankheiten am ehesten mit einer Therapieänderung assoziiert. Ebenso waren eine bestehende Therapie mit Rituximab sowie die Komorbidität einer malignen Erkrankung ein Prädiktor für eine Therapiedeeskalation (Tab. 5).

**Table 5:** 

Faktor	Odds-Ratio (95 %-Konfidenzintervall)	*p*
*Modell A. Rheumaerkrankung*		
GPA/MPA	2,7 (1,2–6,2)	0,022
IgG4-assoziierte Erkrankung	18,0 (3,3–97,8)	0,001
*Modell B. DMARD-Medikation und Prednisolon-Dosis ≥10* *mg/Tag*		
Rituximab-Therapie	4,0 (1,9–8,3)	<0,001
*Model C. Komorbiditäten*		
Alter (pro Jahr)	1,0 (0,9–1,1)	0,099
Diabetes mellitus	0,2 (0,4–1,1)	0,062
Arterielle Hypertonie	2,1 (1,0–4,6)	0,067
Maligne Erkrankung	3,5 (1,3–9,8)	0,017
Kardiovaskuläre Erkrankung	0,3 (0,1–0,9)	0,037

R2 (Modell A) = 0,13; R2 (Modell B) = 0,08; R2 (Modell C) = 0,12

Variablen in Modell A: rheumatoide Arthritis, Granulomatose mit Polyangiitis (GPA)/mikroskopische Polyangiitis (MPA), systemischer Lupus erythematodes (mit oder ohne Antiphospholipidsyndrom), Spondylarthritis/Psoriasisarthritis, eosinophile Granulomatose mit Polyangiitis, IgG4-assoziierte Erkrankung (IgG4), Systemsklerose

Variablen in Modell B: Prednisolon-Dosis ≥10 mg/Tag, csDMARD inklusive HCQ-Monotherapie, Rituximab-Therapie (RTx), TNF-Inhibitortherapie (TNFi), b/tsDMARD-Therapie ohne RTx/TNFi-Medikation, Kombinationstherapie aus csDMARD und b/tsDMARD

Variablen in Modell C: Alter, Nikotinabusus jemals, Asthma bronchiale, chronisch obstruktive Lungenerkrankung, pulmonalarterielle Hypertonie, Diabetes mellitus, arterielle Hypertonie, kardiovaskuläre Erkrankung, zerebrovaskuläre Erkrankung, infektiologische Erkrankung, maligne Erkrankung, chronische Nierenerkrankung, Osteoporose/Osteopenie

## Diskussion

Die abwesende SARS-CoV-2-Infektionsinzidenz in unserer Kohorte deutet darauf hin, dass Patienten mit rheumatologischen Erkrankungen mit und ohne immunmodulatorische Therapie wahrscheinlich kein überproportional erhöhtes Risiko für eine SARS-CoV-2-Infektion haben. Zumindest lag keine überproportionale Infektionshäufigkeit von Rheumapatienten im Vergleich zur Allgemeinbevölkerung vor, welche in Bayern bzw. Oberbayern im beobachteten Zeitraum bei 50.919 bzw. 23.009 (Stand 31.07.2020) lag [10]. Eine abschließende Beurteilung dieser Frage kann jedoch mit unseren Daten aufgrund des fehlenden Nachweises von nachgewiesenen COVID-19-Erkrankungen nicht erfolgen. Ebenso kann über das Risiko eines schweren Verlaufs keine Aussage getroffen werden. Eine weitere Möglichkeit für die niedrige Infektionsinzidenz ist jedoch in Betracht zu ziehen. Viele unserer Patienten berichteten uns von einem ausgeprägten sozialen Rückzug und einer peniblen Einhaltung der Abstands- und Hygieneempfehlungen. Einschränkend ist zu erwähnen, dass keine systematische Erfassung dieser mündlichen Berichte erfolgte, sodass keine objektive Aussage diesbezüglich getroffen werden kann. Daten aus den Niederlanden untermauern jedoch unsere Vermutung, dass sich Patienten mit rheumatischen Erkrankungen deutlich strenger als die Allgemeinbevölkerung an Hygienemaßnahmen halten [11].

Im deutschen COVID-19-Rheuma-Register [7] waren Stand 21.06.2020 *n* = 280 Patienten in ganz Deutschland mit positiver SARS-CoV-2-PCR eingeschlossen [12]. Trotz der Abwesenheit von bestätigten SARS-CoV-2-Infektionen bestand in unserer Kohorte bei 2 % der Patienten der Verdacht auf eine Infektion. Zwar konnte bei diesen Patienten bisher keine Infektion nachgewiesen werden, es ist jedoch zu erwähnen, dass eine serologische Testung erst im April/Mai zur Verfügung stand und dass im Rahmen unserer reinen Verlaufsbeobachtung eine Testung initial nur bei klinisch symptomatischen Patienten durchgeführt wurde. Der Anteil dieser Verdachtsfälle in unserer Kohorte ist vergleichbar mit Daten bestätigter SARS-CoV-2-positiver Rheumapatienten aus anderen Kohorten [13–15].

Erste Daten der internationalen Studien der COVID-19 Global Rheumatology Alliance deuten darauf hin, dass ein Fortführen der meisten immunsuppressiven Therapien relativ sicher zu sein scheint [4, 5]. Zu beachten gilt, dass v. a. eine mittlere bis hohe Glukokortikoiddosis (äquivalent ≥10 mg Prednisolon/Tag) mit einem höheren Risiko für eine Hospitalisierung aufgrund von COVID-19 assoziiert war, wohingegen DMARDs nicht mit einem erhöhten Risiko assoziiert waren [5]. Unsere Strategie, bei Patienten v. a. mit ANCA-assoziierten Vaskulitiden und IgG4-assoziierten Erkrankungen in stabiler Remission auf eine weitere Gabe von Rituximab zu verzichten, spiegelt sich auch retrospektiv in den Ergebnissen der logistischen Regression wider. Hierbei stellten sich eine Rituximab-Therapie und eine Erkrankung mit ANCA-assoziierter Vaskulitis oder IgG4-assoziierten Erkrankungen als Determinanten einer Therapiepause dar. Dieses Vorgehen deckt sich mit aktuellen Vermutungen, dass eine B‑Zell-Depletion durch Rituximab mit schwereren Verläufen einhergehen könnte [16], obwohl diese Frage sicherlich erst durch größere Fallzahlen in den Registern beantwortet werden muss. Da Rituximab v. a. bei GPA/MPA und IgG4-assoziierter Erkrankung eingesetzt wurde, zeigten sich diese Erkrankungen ebenfalls mit einer Therapiereduktion assoziiert. Gerade bei der GPA/MPA und Beteiligung des respiratorischen Trakts besteht die Sorge eines schweren Verlaufs unter Rituximab-Therapie [17]. Vor Erhalt der ersten Daten zur DMARD-Medikation kam es im Rahmen eines „shared decision making“ zu Therapiereduktion bei 15 % unserer Patienten. Das Anliegen einer Therapiepause wurde von mehr als diesen 15 % vorgetragen aus Sorge vor schweren Verläufen einer Infektion. Bei einem Teil dieser Patienten stellte sich der Verweis auf die DGRh-Empfehlungen als hilfreich dar [9, 18]. Bei 7 Patienten wurde eine TNF-Inhibitortherapie aufgrund der Pandemie pausiert oder beendet. Im Lichte jüngerer Daten, die eine leicht erniedrigte Odds-Ratio für eine Hospitalisierung bei der Einnahme von TNF-Inhibitoren zeigen [5], würden wir speziell bei dieser bDMARD-Klasse besonders von einer Therapiepause abraten.

Zumindest im kurzfristigen Verlauf von 5 Beobachtungsmonaten führte die Therapiereduktion bei den meisten Patienten nicht zu einem Schub der Grunderkrankung, weshalb ein solches Vorgehen je nach Erkrankung, Therapiephase und Vulnerabilität für einen schweren COVID-19-Verlauf im Einzelfall gerechtfertigt sein kann.

Eine logistische Regression zur retrospektiven Evaluation unserer Entscheidungen erbrachte zudem eine erhöhte Odds-Ratio bei einer malignen Erkrankung für eine Therapiereduktion sowie zu einer eher zurückhaltenden Entscheidung zur Therapiedeeskalation, wenn eine kardiovaskuläre Erkrankung vorlag.

Folgende Limitationen der Studie sollten berücksichtigt werden. Aufgrund der fehlenden Inzidenz von SARS-CoV-2-Infektionen konnten die primären Analysen und die sekundäre Analyse zur Schwere des Verlaufs nicht durchgeführt werden. Wir fokussierten uns daher auf eine Darstellung unserer Rheumakohorte unter Pandemiebedingungen sowie die Analyse von pandemiebedingten Therapieentscheidungen. Zwar wurden Patienten dazu angehalten, sich bei Verdacht auf eine SARS-CoV-2-Infektion umgehend in unserer Ambulanz zu melden, und zudem im Rahmen ihrer mindestens 12-wöchentlichen Verlaufskontrollen auf Symptome hin befragt. Eine ausgebliebene Infektionsmeldung können wir jedoch nicht mit absoluter Sicherheit ausschließen. Aufgrund der Spezialisierung auf Systemerkrankungen mit potenzieller Nierenbeteiligung (v. a. ANCA-Vaskulitiden und SLE) in unserer Ambulanz zeigen sich bei der Krankheitsverteilung Unterschiede zur üblichen Häufigkeitsverteilung einer rheumatologischen Ambulanz oder Praxis. Zudem kann wegen der geringen Größe der Kohorte und der fehlenden Fälle kein Rückschluss auf ein möglicherweise erhöhtes Infektionsrisiko im Vergleich zur gesunden Bevölkerung gezogen werden. Wir können lediglich anhand unserer Beobachtung mutmaßen, dass das Risiko zumindest nicht exorbitant erhöht zu sein scheint. Zu guter Letzt lässt die kleine Anzahl an Patienten, die nach Absetzen der jeweiligen immunmodulatorischen Therapie einen Flare entwickelten, keine Rückschlüsse auf die Sicherheit des Absetzens der jeweiligen Medikation zu. Das werden größere Registerdaten beantworten müssen.

Zusammenfassend zeigen unsere Beobachtungen, dass im Vergleich zur Allgemeinbevölkerung zumindest keine überproportional erhöhte Inzidenz einer SARS-CoV-2-Infektion bei rheumatologischen Patienten in einem Hotspot-Gebiet zu bestehen scheint, was eine gewisse Zuversicht für einen künftigen Wiederanstieg der Infektionszahlen schafft. Insbesondere für die medikamentöse Therapie bedeutet das, dass ein Pausieren oder eine Reduktion der Medikation nicht sinnvoll erscheint und erst im Falle einer Infektion erfolgen sollte.

## Fazit für die Praxis

- Die erste Welle der COVID-19-Pandemie stellte für die ambulante Versorgung von Rheumapatienten eine besondere Herausforderung dar.
- In dieser bayrischen Single-Center-Kohorte legt das gänzliche Fehlen von bestätigten COVID-19 Fällen in einer sonst relativ stark betroffenen Region den Schluss nahe, dass kein überproportional erhöhtes Infektionsrisiko für unsere rheumatologischen Patienten zu bestehen scheint.
- Eine Fortführung der meisten immunsuppressiven Therapien scheint daher unter Berücksichtigung der Infektionsschutzmaßnahmen legitim und sinnvoll.

## References

[1] Guan WJ, Ni ZY, Hu Y, Liang WH, Ou CQ, He JX, Liu L, Shan H, Lei CL, Hui DSC, Du B, Li LJ, Zeng G, Yuen KY, Chen RC, Tang CL, Wang T, Chen PY, Xiang J, Li SY, Wang JL, Liang ZJ, Peng YX, Wei L, Liu Y, Hu YH, Peng P, Wang JM, Liu JY, Chen Z, Li G, Zheng ZJ, Qiu SQ, Luo J, Ye CJ, Zhu SY, Zhong NS (2020). Clinical characteristics of coronavirus disease 2019 in China. N Engl J Med.

[2] Wang D, Hu B, Hu C, Zhu F, Liu X, Zhang J, Wang B, Xiang H, Cheng Z, Xiong Y, Zhao Y, Li Y, Wang X, Peng Z (2020). Clinical characteristics of 138 hospitalized patients with 2019 novel coronavirus-infected pneumonia in Wuhan, China. JAMA.

[3] Huang C, Wang Y, Li X, Ren L, Zhao J, Hu Y, Zhang L, Fan G, Xu J, Gu X, Cheng Z, Yu T, Xia J, Wei Y, Wu W, Xie X, Yin W, Li H, Liu M, Xiao Y, Gao H, Guo L, Xie J, Wang G, Jiang R, Gao Z, Jin Q, Wang J, Cao B (2020). Clinical features of patients infected with 2019 novel coronavirus in Wuhan, China. Lancet.

[4] Putman M, Chock YPE, Tam H, Kim AHJ, Sattui SE, Berenbaum F, Danila MI, Korsten P, Sanchez-Alvarez C, Sparks JA, Coates LC, Palmerlee C, Peirce A, Jayatilleke A, Johnson SR, Kilian A, Liew J, Prokop LJ, Murad MH, Grainger R, Wallace ZS, Duarte-García A (2020). Antirheumatic disease therapies for the treatment of COVID-19: a systematic review and meta-analysis. Arthritis Rheumatol.

[5] Gianfrancesco M, Hyrich KL, Al-Adely S, Carmona L, Danila MI, Gossec L, Izadi Z, Jacobsohn L, Katz P, Lawson-Tovey S, Mateus EF, Rush S, Schmajuk G, Simard J, Strangfeld A, Trupin L, Wysham KD, Bhana S, Costello W, Grainger R, Hausmann JS, Liew JW, Sirotich E, Sufka P, Wallace ZS, Yazdany J, Machado PM, Robinson PC (2020). Characteristics associated with hospitalisation for COVID-19 in people with rheumatic disease: data from the COVID-19 Global Rheumatology Alliance physician-reported registry. Ann Rheum Dis.

[6] Gianfrancesco MA, Hyrich KL, Gossec L, Strangfeld A, Carmona L, Mateus EF, Sufka P, Grainger R, Wallace Z, Bhana S, Sirotich E, Liew J, Hausmann JS, Costello W, Robinson P, Machado PM, Yazdany J (2020). Rheumatic disease and COVID-19: initial data from the COVID-19 Global Rheumatology Alliance provider registries. Lancet Rheumatol.

[7] Hasseli R, Mueller-Ladner U, Schmeiser T, Hoyer BF, Krause A, Lorenz HM, Regierer AC, Richter JG, Strangfeld A, Voll RE, Pfeil A, Schulze-Koops H, Specker C (2020). National registry for patients with inflammatory rheumatic diseases (IRD) infected with SARS-CoV-2 in Germany (ReCoVery): a valuable mean to gain rapid and reliable knowledge of the clinical course of SARS-CoV-2 infections in patients with IRD. RMD Open.

[8] Zheng Z, Peng F, Xu B, Zhao J, Liu H, Peng J, Li Q, Jiang C, Zhou Y, Liu S, Ye C, Zhang P, Xing Y, Guo H, Tang W (2020). Risk factors of critical & mortal COVID-19 cases: a systematic literature review and meta-analysis. J Infect.

[9] Schulze-Koops H, Holle J, Moosig F, Specker C, Aries P, Burmester G, Fiehn C, Hoyer B, Krause A, Leipe J, Lorenz HM, Schneider M, Sewerin P, Voormann A, Wager U, Krüger K, Iking-Konert C (2020). Current guidance of the German Society of Rheumatology for the care of patients with rheumatic diseases during the SARS-CoV-2/Covid 19 pandemic. Z Rheumatol.

[10] Bayerisches Landesamt für Gesundheit und Lebensmittelsicherheit (2020) Übersicht der Fallzahlen von Coronavirusinfektionen in Bayern. https://www.lgl.bayern.de/gesundheit/infektionsschutz/infektionskrankheiten_a_z/coronavirus/karte_coronavirus/index.htm. Zugegriffen: 31.07.2020

[11] Hooijberg F, Boekel L, Vogelzang EH, Leeuw M, Boers M, van Vollenhoven R, Lems WF, Nurmohamed MT, Wolbink G (2020). Patients with rheumatic diseases adhere to COVID-19 isolation measures more strictly than the general population. Lancet Rheumatol.

[12] Hasseli R, Müller-Ladner U, Hoyer BF, Krause A, Lorenz HM, Pfeil A, Richter JG, Schmeiser T, Schulze-Koops H, Strangfeld A, Voll RE, Specker C, Regierer AC (2020). Alter, Komorbiditäten und Therapie mit Glukokortikoiden sind Risikofaktoren für eine COVID-19-bedingte Hospitalisierung: erste Ergebnisse aus dem deutschen COVID-19-Rheuma-Register.

[13] Simon D, Tascilar K, Krönke G, Kleyer A, Zaiss M, Heppt F, Meder C, Atreya R, Klenske E, Dietrich P, Abdullah A, Kliem T, Corte G, Leppkes M, Kremer A, Ramming A, Pachowsky M, Schuch F, Ronneberger M, Kleinert S, Maier C, Hueber AJ, Manger K, Manger B, Berking C, Tenbusch M, Überla K, Sticherling M, Neurath MF, Schett G (2020). Patients with immune-mediated inflammatory diseases receiving cytokine inhibitors have low prevalence of SARS-CoV-2 seroconversion.

[14] Andreica I, Kiefer D, Rezniczek GA, Kiltz U, Jast R, Buehring B, Baraliakos X, Braun J (2020). Patienten mit entzündlich-rheumatischen Erkrankungen unter immunsuppressiver Therapie wechselten häufig die Therapie, waren aber nur selten SARS-CoV-2 infiziert.

[15] Aries P, Iking-Konert C (2020). No increased rate of SARS-CoV-2 infection for patients with inflammatory rheumatic diseases compared with the general population in the city of Hamburg (Germany). Ann Rheum Dis.

[16] Schulze-Koops H, Krueger K, Vallbracht I, Hasseli R, Skapenko A (2020). Increased risk for severe COVID-19 in patients with inflammatory rheumatic diseases treated with rituximab. Ann Rheum Dis.

[17] Guilpain P, Le Bihan C, Foulongne V, Taourel P, Pansu N, Maria ATJ, Jung B, Larcher R, Klouche K, Le Moing V (2020). Rituximab for granulomatosis with polyangiitis in the pandemic of covid-19: lessons from a case with severe pneumonia. Ann Rheum Dis.

[18] Schulze-Koops H, Iking-Konert C, Leipe J, Hoyer BF, Holle J, Moosig F, Aries P, Burmester G, Fiehn C, Krause A, Lorenz HM, Schneider M, Sewerin P, Voormann A, Wagner U, Krüger K, Specker C (2020). Recommendations of the German Society for Rheumatology for management of patients with inflammatory rheumatic diseases in the context of the SARS-CoV-2/COVID-19 pandemic—update July 2020. Z Rheumatol.

